# The impact of diabetes status on pain and physical function following total joint arthroplasty for hip and knee osteoarthritis: variation by sex and body mass index

**DOI:** 10.1038/s41598-024-61847-0

**Published:** 2024-05-15

**Authors:** J. Denise Power, Anthony V. Perruccio, Mayilee Canizares, J. Roderick Davey, Rajiv Gandhi, Nizar N. Mahomed, Khalid Syed, Christian Veillette, Y. Raja Rampersaud

**Affiliations:** 1 Schroeder Arthritis Institute, Krembil Research Institute, University Health Network, 399 Bathurst Street MP10-326, Toronto, ON M5T 2S8 Canada; 2 Arthritis Community Research & Epidemiology Unit, Toronto, ON Canada; 3 Orthopaedics, Department of Surgery, University of Toronto, Toronto, ON Canada; 4 Institute of Health Policy, Management and Evaluation, University of Toronto, Toronto, ON Canada; 5 ICES, Toronto, ON Canada; 6 Neurosurgery, Department of Surgery, University of Toronto, Toronto, ON Canada

**Keywords:** Health care, Risk factors

## Abstract

Few studies have examined diabetes impact on total joint arthroplasty (TJA) outcomes, with variable findings. We investigated the association between diabetes and post-TJA physical function and pain, examining whether diabetes impact differs by sex and BMI. Patient sample completed questionnaires within 3 months prior to hip or knee TJA for osteoarthritis (OA) and 1-year post-surgery. Surgical ‘non-response’ was defined as < 30% improvement in Western Ontario and McMaster Universities Osteoarthritis Index (WOMAC) pain and physical function at 1-year. Two adjusted logistic regression models were estimated: (1) excluding, (2) including an interaction between diabetes, sex and BMI. The sample (626 hip, 754 knee) was 54.9% female, had mean BMI of 30.1, 13.0% reported diabetes. In adjusted models excluding an interaction, diabetes was not associated with non-response. However, a significant 3-way interaction (physical function: p = 0.003; pain: p = 0.006) between diabetes, sex, and BMI was found and was associated with non-response: non-response probability increased with increasing BMI in men with diabetes, but decreased with increasing BMI in women in diabetes. Findings suggest uncertainty in diabetes impact may be due to differential impacts by sex and BMI. A simple consideration of diabetes as present vs. absent may not be sufficient, with implications for the large TJA population.

## Introduction

Hip and knee total joint arthroplasties (TJA) are cost-effective treatments for end-stage osteoarthritis (OA) and are among the most frequently performed elective surgical procedures^1,2^. They represent the majority of direct expenditures for OA and demand for these surgeries is expected to increase with the aging of the population^2,3^. Despite their relatively high effectiveness, up to one-third of TJA patients continue to report residual symptoms such as pain and disability after surgery^4^. Given the high and increasing volume of these procedures, the individual and population impact of poor TJA outcomes is large and costly. Research directed at identifying and understanding factors associated with poor TJA outcomes is both timely and highly relevant.

Like OA, diabetes is an increasingly prevalent chronic condition globally and is a common comorbidity in people with OA. A 2015 meta-analysis^5^ found that the risk of prevalent diabetes was 1.4 times greater for those with vs. without OA, yielding an estimated prevalence of 14.4% among OA patients. Diabetes is associated with a number of adverse effects that may be relevant to TJA outcomes, including decreased wound healing, reduced muscle strength and joint mobility, and impaired bone quality^6,7^. Hyperglycemia can lead to chronic systemic inflammation that impacts the entirety of the body, including the joints. It can also lead to the production of advanced glycation end products that can accumulate in the joints, increasing cartilage stiffness and bone fragility^8^.

Diabetes has been associated with higher rates of TJA complications, primarily infections and aseptic implant loosening^9^. However, its broader impact on TJA patient reported outcome measures (PROMs) is less clear, as reported findings have been quite variable^10–14^. In most research examining the impact of diabetes in OA, sex has been considered as a confounding, and thus an adjustment, variable. However, given known sex differences in OA, TJA and diabetes, a simple adjustment may be insufficient to determine impact.

Studies have consistently identified sex differences in OA. Prevalence and incidence is higher in females than males, and females have more clinical pain, inflammation, physical difficulty and impaired joint function^15,16^. In addition, women tend to have worse symptoms at the time of TJA surgery for OA. This likely contributes to the finding that women have poorer physical function and greater pain levels after surgery, although they experience similar or greater improvement than men relative to pre-surgery^16,17^. Relatively little research, however, has been directed at examining whether risk factors for a poor TJA outcome may be sex-specific. This may be particularly relevant for understanding the impact of comorbid diabetes in patients with OA undergoing TJA, as diabetes itself is associated with a number of notable sex differences.

There are known variations in risk, pathophysiology and complications of diabetes by sex. Women have a higher prevalence of diabetes at older ages, and women with diabetes are at greater risk of macrovascular complications (e.g. myocardial infarction, stroke), whereas men with diabetes are a greater risk of microvascular complications (e.g. neuropathy, nephropathy)^18,19^. Additionally, there is evidence to suggest that the effects of obesity among people with diabetes, may differ by sex^20^. However, while body mass index (BMI) is typically considered an important control variable in analyses examining the effects of diabetes on outcomes, sex variation in its effects are not often considered. Using data from the NHANES III, Karvonen-Gutierrez et al.^20^ found cardio-metabolic risk factors for knee OA differed by sex and obesity status. In both obese and non-obese men, insulin resistance was related to knee OA. In non-obese women, the association was much weaker, while in obese women, insulin resistance was inversely related to knee OA. These findings suggest that the metabolic or inflammatory impacts of obesity and diabetes and/or their roles in OA may also differ by sex.

We hypothesized that the variability in findings in the literature on the impact of diabetes on TJA outcomes may have been influenced by a lack of consideration of the potential for sex and BMI to differentially affect diabetes impact. The purpose of our study was to examine the association between diabetes and post-TJA pain and physical function in hip and knee OA patients, explicitly examining whether diabetes impact differs by sex and BMI. To the best of our knowledge, this is the first study to consider diabetes impact in a TJA population in this manner. Findings may aid in clarifying the variability in findings in the literature and may have important implications for pre- and peri-operative TJA patient education and management.

## Methods

The current analysis utilizes data from a prospective cohort study (Longitudinal Evaluation in the Arthritis Progra—LEAP-OA) examining factors affecting outcomes following TJA for OA conducted at the Toronto Western Hospital in Toronto, Canada. Patients with end-stage hip or knee OA scheduled for TJA were consecutively recruited from Nov 2015 to Dec 2018. Eligibility criteria included ≥ 35 years of age and the ability to read and comprehend English. Individuals undergoing revision procedures and those with post-traumatic or inflammatory arthritis were excluded. Participants were 626 hip and 754 knee OA patients scheduled for unilateral TJA. The study was approved by the University Health Network Research Ethics Board (16-5759). Written informed consent was obtained from all patients.

### Data collection

Patients completed a questionnaire within the 3 weeks prior to surgery at their pre-surgical appointment and a follow-up questionnaire at their 12-month post-surgical clinical visit. Collecting data during these routinely scheduled appointments was selected to ensure that patients were at similar time points in terms of their pre-surgical symptom state, as well as length of time from pre-surgical to one year post-surgical time point.

#### Socio-demographic variables

Data on socio-demographic characteristics were collected in the pre-surgery questionnaire, including sex, age and highest level of education (≤ High school vs. post-secondary).

#### Pre-surgery health-related characteristics variables

A comorbidity-related count variable was derived from yes/no responses to an extended list of 18 conditions (high blood pressure, lung disease, ulcer/stomach disease, kidney disease, liver disease, anemia/other blood disease, cancer, depression, coronary heart disease/heart attack, heart failure, stroke, high cholesterol, thyroid condition, sleep apnea, dementia, migraine, chronic pelvic pain, fibromyalgia), based on the American Academy of Orthopedic Surgeon’s Comorbidity scale and excluding diabetes^21^. A separate variable for diabetes status (present vs. absent) was created.

Data on measured height and weight were used to compute body mass index (BMI). Participants indicated on a homunculus diagram any joints/sites that were affected by arthritis and were painful on most days for at least a month. A summed count score of left and right affected joints was derived, excluding the surgical joint/site, with possible scores ranging from 0 to 19.

Depressive and anxiety symptoms were measured using the Hospital Anxiety and Depression Scale (HADS)^22^. This measure has been found to be a reliable and valid measure for assessing emotional distress in medical populations^23^ and provides separate scores for depressive and anxiety symptoms ranging from 0 to 21. Higher scores indicate greater symptoms.

A dichotomous variable for opioid pain medication use was created, daily/occasional use vs. no use.

Neuropathic pain symptoms were assessed using the painDETECT questionnaire (PD-Q)^24^, which consists of 9 items that evaluate pain quality, pattern and radiation. Possible scores range from -1 to 38, with higher scores indicating more neuropathic-like symptoms. Sensitivity, specificity and predictive accuracy of 80–84% were determined in a heterogeneous group of pain patients relative to pain physicians’ clinical assessments. The PD-Q has been used in a number of clinical populations, including knee OA and other musculoskeletal conditions, with favourable reliability and validity^25^. For the current study, patients were prompted to consider their hip or knee pain, as appropriate. Scores were dichotomized as unlikely/possibly vs. likely neuropathic pain (scores: ≤ 18 vs. ≥ 19)^24^.

#### Physical function

Data on pre- and post-surgical physical function limitations were captured using the 17-item physical function subscale of the Western Ontario McMaster University Osteoarthritis Index (WOMAC), which measures the degree of difficulty when performing daily activities. This subscale is widely used in OA and TJA and has favourable measurement properties^26,27^. Possible scores range from 0 to 68, with higher scores indicating greater physical function limitations.

#### Pain

Data on pre- and post-surgical pain were captured using the WOMAC pain subscale, which assess hip or knee pain during the past week in five different situations^26^. This measure is the most commonly used patient-reported lower extremity pain measure in OA and TJA and has favourable reliability and validity^27^. Scores range from 0 to 20, with higher scores indicating greater pain severity.

##### Study outcome variables

Our two primary outcomes of interest were patient-reported surgical ‘non-response’ in (1) physical function and (2) pain, defined as < 30% improvement in the corresponding WOMAC subscale by 12-months post-surgery relative to pre-surgery. There are a number of WOMAC-based responder criteria that have been established using various methodologies. We focussed on percentage change, as WOMAC-based responder criteria relying on absolute change have been found to vary by baseline score^28,29^, and literature suggests that percentage change estimates may be generally preferable, particularly for patients with low or high pre-surgery scores^30^. A thirty percent change has been shown to be a meaningful improvement in pain studies of various chronic pain populations^31,32^ and has been characterized as representing ‘moderately important’ improvement according to the IMMPACT recommendations for chronic pain clinical trials^30^. Further, Conaghan et al.^33^ determined that meaningful within person change of the WOMAC subscales in patients with moderate to severe hip or knee OA ranged from 25 to 32% for a 2-category change in patient global assessment.

### Analyses

Descriptive statistics were generated for all variables; means and standard deviations for continuous variables, frequencies and percentages for categorical variables. These were generated overall and separately for patients with and without self-reported diabetes. Differences between these groups were assessed using t-tests and chi-square tests, as appropriate. Rates (percentages and 95% confidence intervals) of surgical non-response in pain and physical function were calculated by diabetes status. Multivariable logistic regressions were used to examine the associations between non-response (outcome variable) and diabetes status, adjusted for each of the above noted study variables. Two models were estimated for each of the two primary outcome variables, one excluding, and the other including, a three-way interaction between diabetes status, sex and BMI. Results for these three variables were depicted graphically to aid interpretation.

#### Supplemental/sensitivity analyses

While < 30% change was our primary responder criteria, we nonetheless considered alternative definitions of non-response in order to evaluate the stability of our findings across responder definitions. First, we used published joint-specific minimal clinically important difference (MCID) criteria^28,29^ for absolute change and defined non-response as a < MCID improvement in WOMAC pain and WOMAC physical function by 12 months post-surgery. We additionally considered non-response as not fulfilling the OARSI-OMERACT definition of response^34^. This definition jointly considers absolute and percentage change in WOMAC pain and physical function. The regression analyses were repeated using these alternate definitions for responder outcome determination.

In addition, an alternate measure of adiposity was considered, pre-surgical waist circumference instead of BMI. Regression analyses using the primary responder criteria were repeated incorporating this change. All analyses were conducted using SAS version 9.4.

## Results

Table 1 describes the study sample and compares the 179 participants (13.0%) who reported diabetes with the 1201 participants (87.0%) who did not.

**Table 1:** Pre-surgery sample characteristics, overall and by diabetes status.

Variable	Mean (SD) or Frequency (%)			p-value*
	Overall sample (N = 1380)	With diabetes (N = 179; 13.0%)	Without diabetes (N = 1201; 87.0%)	
Surgical site				** < 0.001**
Knee	754 (54.6%)	121 (67.6%)	633 (52.7%)	
Hip	626 (45.4%)	58 (32.4%)	568 (47.3%)	
Sex				0.832
Female	758 (54.9%)	97 (54.2%)	661 (55.0%)	
Male	622 (45.1%)	82 (45.8%)	540 (45.0%)	
Age Mean (SD)	65.5 (9.3)	66.7 (7.6)	65.4 (9.5)	**0.038**
Education				**0.021**
≤ High school	395 (29.3%)	63 (36.8%)	332 (28.2%)	
Post-secondary	952 (70.7%)	108 (63.2%)	844 (71.8%)	
BMI mean (SD)	30.1 (6.2)	32.2 (6.3)	29.8 (6.1)	** < 0.001**
Comorbidity count mean (SD)	1.8 (1.6)	2.7 (2.2)	1.6 (1.5)	** < 0.001**
Symptomatic joint count mean (SD)	2.8 (3.1)	2.7 (3.4)	2.8 (3.1)	0.787
Depressive Symptoms score (0 to 21) mean (SD)	5.1 (3.6)	5.6 (3.9)	5.1 (3.5)	0.058
Anxiety Symptoms score (0 to 21) mean (SD)	5.3 (4.0)	5.6 (4.5)	5.2 (3.9)	0.314
WOMAC pain score (0 to 20) mean (SD)	10.3 (3.7)	11.1 (4.2)	10.2 (3.6)	**0.014**
WOMAC physical function score (0 to 68) mean (SD)	36.8 (12.6)	38.4 (13.9)	36.5 (12.3)	0.079
Opioid use				0.208
No use	948 (71.7%)	115 (67.6%)	833 (72.3%)	
Daily or occasional	374 (28.3%)	55 (32.4%)	319 (27.7%)	
Neuropathic pain score (− 1 to 38)				0.129
Unlikely or possibly	1128 (87.3%)	138 (83.6%)	990 (87.8%)	
Likely	164 (12.7%)	27 (16.4%)	137 (12.2%)	

*p-value for comparing with vs. without diabetes.

Significant values are in bold.

Over half (54.6%) of the sample were undergoing knee TJA. This proportion was significantly higher for those with diabetes (67.6%) than those without (52.7%; p < 0.001). Approximately 55% of the sample was female. Mean age was 65.5 years overall, and was higher among those who reported diabetes (66.7 years) than those who did not (65.4 years; p = 0.038). Mean BMI was also significantly higher for those with diabetes (32.2 vs. 29.8; p < 0.001), as was the mean number of additional chronic conditions reported (2.7 vs. 1.6; p < 0.001). Comorbidity count ranged from 0 to 11 for those with diabetes and 0 to 8 for those without diabetes (range data not tabulated). Symptomatic joint count did not vary for those with and without diabetes, with no significant difference in means (p = 0.787) and counts ranging from 0 to 17 and 0 to 19, for those with and without diabetes, respectively. Baseline mean WOMAC pain (11.1 vs. 10.2) and physical function (38.4 vs. 36.5) scores were higher for those with diabetes, although these differences were only statistically significant for pain (pain: p = 0.014; physical function: p = 0.079). A notable proportion of the sample (12.7%) reported symptoms consistent with likely neuropathic pain. This proportion was 16.4% among those with diabetes and 12.2% among those without diabetes (p = 0.129).

Mean age for females was 65.6 years (64.9, 66.2) and for males was 65.5 years (64.7, 66.2) (p = 0.846) (data not tabulated). Mean BMI for females and males was 30.3 (95% CI 29.8, 30.8) and 29.9 (95% CI 29.5, 30.3), respectively (p = 0.233). BMI ranged from 18.3 to 55.0 for females and 18.5 to 58.8 for males. The percentage of patients with diabetes did not vary by sex (p = 0.832), with 12.8% (95% CI 10.4%, 15.2%) of females and 13.2% (95% CI 10.5%, 15.8%) of males indicating they had been diagnosed with the disease.

Rates of surgical non-response defined as experiencing a less than 30% improvement in WOMAC scores are presented in Table 2, overall and by diabetes status, for pain and physical function. Physical function non-response rates were not significantly different (p = 0.107) between those with (18.3%; 95% CI 12.2%, 24.4%) and without diabetes (13.4%; 95% CI 11.4%, 15.5%). However, non-response rates for pain were significantly higher (p = 0.001) for those with diabetes [(19.2%; 95% CI 12.9%, 25.4%) vs. (10.2%; 95% CI 8.3%, 12.0%)].

**Table 2:** Non-response rates* overall and by diabetes status.

	Percentage (95% Confidence Interval)	
	Physical function non-response	Pain non-response
Overall	14.1% (12.1%, 16.1%)	11.3% (9.5%, 13.1%)
With Diabetes	18.3%** (12.2%, 24.4%)	19.2%^†^ (12.9%, 25.4%)
Without Diabetes	13.4% (11.4%, 15.5%)	10.2% (8.3%, 12.0%)

*Non-response at 12 months post-surgery was defined as < 30% improvement in score relative to pre-surgery.

Comparing with and without diabetes: **p = 0.107; ^†^p = 0.001.

**Figure 1:**
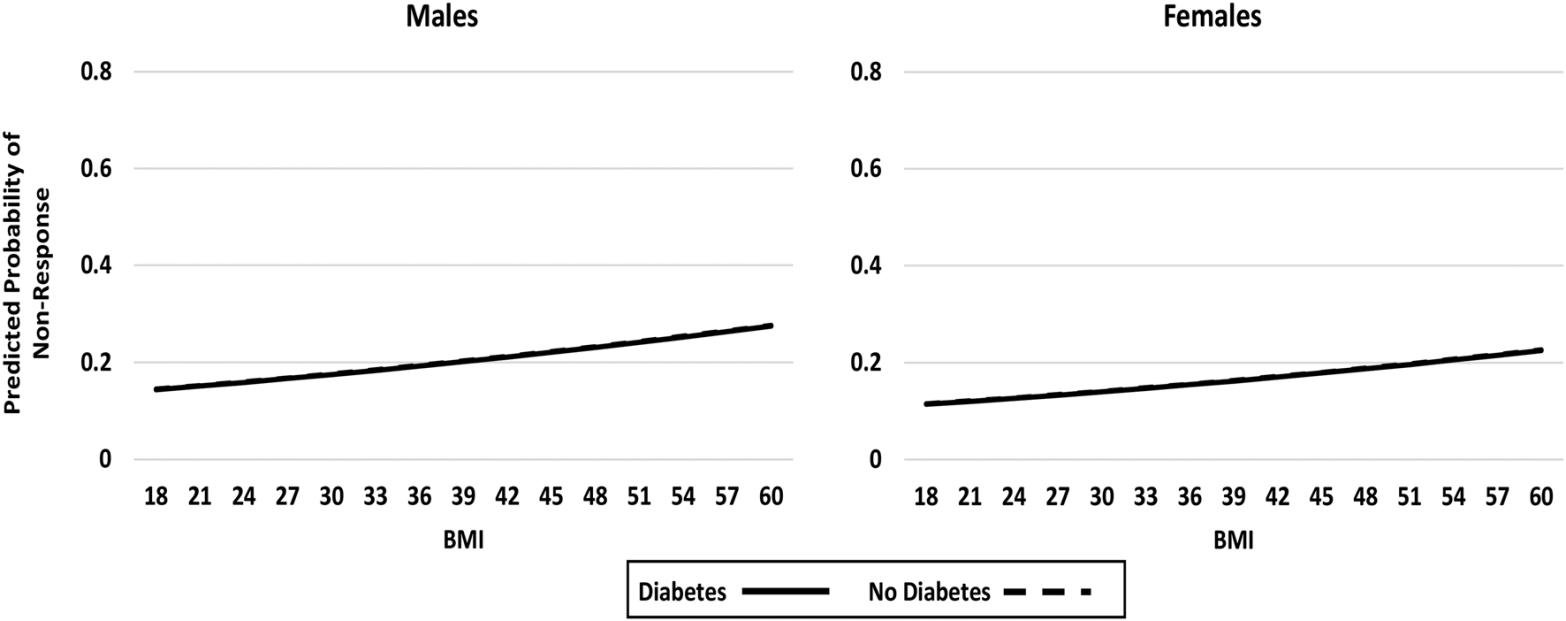
Impact of diabetes status, sex and BMI on post-surgical WOMAC physical function non-response (< 30% improvement in WOMAC physical function); Model 1—no interaction (showing no significant effects by sex, BMI or diabetes status).

**Figure 2:**
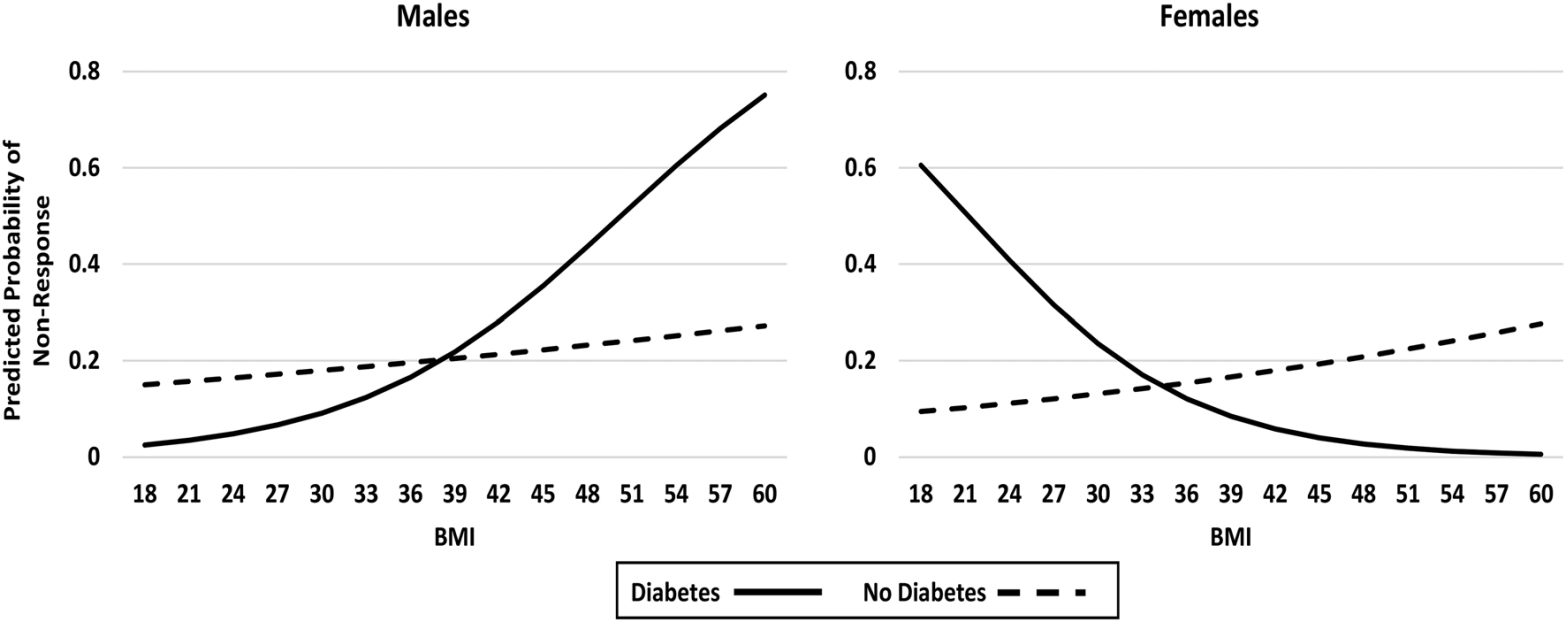
Impact of diabetes status, sex and BMI on post-surgical physical function non-response (< 30% improvement in WOMAC physical function); Model 2—with interactions (showing differential diabetes effects by sex and BMI).

In multivariable regression analyses with physical function non-response as the dependant variable, diabetes was not significantly associated (p = 0.970) with non-response in the initial model that considered the independent effect of diabetes status controlling for sex, BMI and the pre-surgical socio-demographic and health status characteristics (Table 3: Model 1 and Fig. 1). In Model 2, with the addition of a three-way interaction allowing for the effect of diabetes to vary by sex and BMI, diabetes had a significant effect on physical function non-response (p = 0.003) (Table 3: Model 2 and Fig. 2). For males with diabetes, increasing BMI was associated with an increased probability of non-response. In contrast, for females with diabetes, increasing BMI was associated with a decreased probability of non-response.

**Table 3:** Predictors of non-response (< 30% improvement) in WOMAC physical function at 12 months post-TJR.

Variable	Model 1: without interaction*		Model 2: with interaction** (DIABETES*BMI*SEX)	
	Odds ratio (95% CI)	p-value	Odds Ratio (95% CI)	p-value
Surgical site (Hip vs. Knee)	**0.37 (0.24, 0.57)**	** < 0.001**	**0.38 (0.25, 0.59)**	** < 0.001**
Sex (Female vs. Male)	0.77 (0.53, 1.12)	0.173	**	0.470
Age	1.01 (0.99, 1.03)	0.513	1.01 (0.99, 1.03)	0.417
Education (Post-secondary vs. ≤ High school)	0.71 (0.48, 1.05)	0.086	0.70 (0.47, 1.04)	0.074
BMI	1.02 (0.99, 1.05)	0.243	**	0.531
Comorbidity Count	1.01 (0.89, 1.14)	0.903	0.99 (0.88, 1.11)	0.891
Symptomatic joint count	**1.06 (1.00, 1.12)**	**0.051**	**1.06 (1.00, 1.12)**	**0.052**
Depressive Symptoms score (0 to 21)	1.06 (0.99, 1.14)	0.073	**1.07 (1.00, 1.15)**	**0.048**
Anxiety Symptoms score (0 to 21)	1.02 (0.97, 1.09)	0.429	1.02 (0.97, 1.09)	0.438
WOMAC pain score (0 to 20)	1.02 (0.92, 1.13)	0.704	1.03 (0.93, 1.14)	0.563
WOMAC physical function score (0 to 68)	**0.97 (0.94, 0.99)**	**0.018**	**0.96 (0.93, 0.99)**	**0.007**
Opioid Use (Daily or occasional vs. No use)	0.83 (0.54, 1.29)	0.408	0.84 (0.54, 1.31)	0.441
Neuropathic pain score (− 1 to 38) (Likely vs. Unlikely/possibly)	**2.42 (1.41, 4.16)**	**0.001**	**2.48 (1.44, 4.28)**	**0.001**
Diabetes (present vs. absent)	0.99* (0.58,1.69)	0.97	**	0.089
Diabetes*BMI	–	–	**	0.122
Diabetes*Sex	–	–	**	**0.002**
Sex*BMI	–	–	**	0.703
Diabetes*Sex*BMI	–	–	**	**0.003**

*See Fig. 1 (Model 1, without interaction) for depiction of the effect of diabetes, sex and BMI.

**To interpret interactions, please refer to Fig. 2 (Model 2, with interaction) for depiction of the effects of diabetes, sex and BMI.

Significant values are in bold.

**Figure 3:**
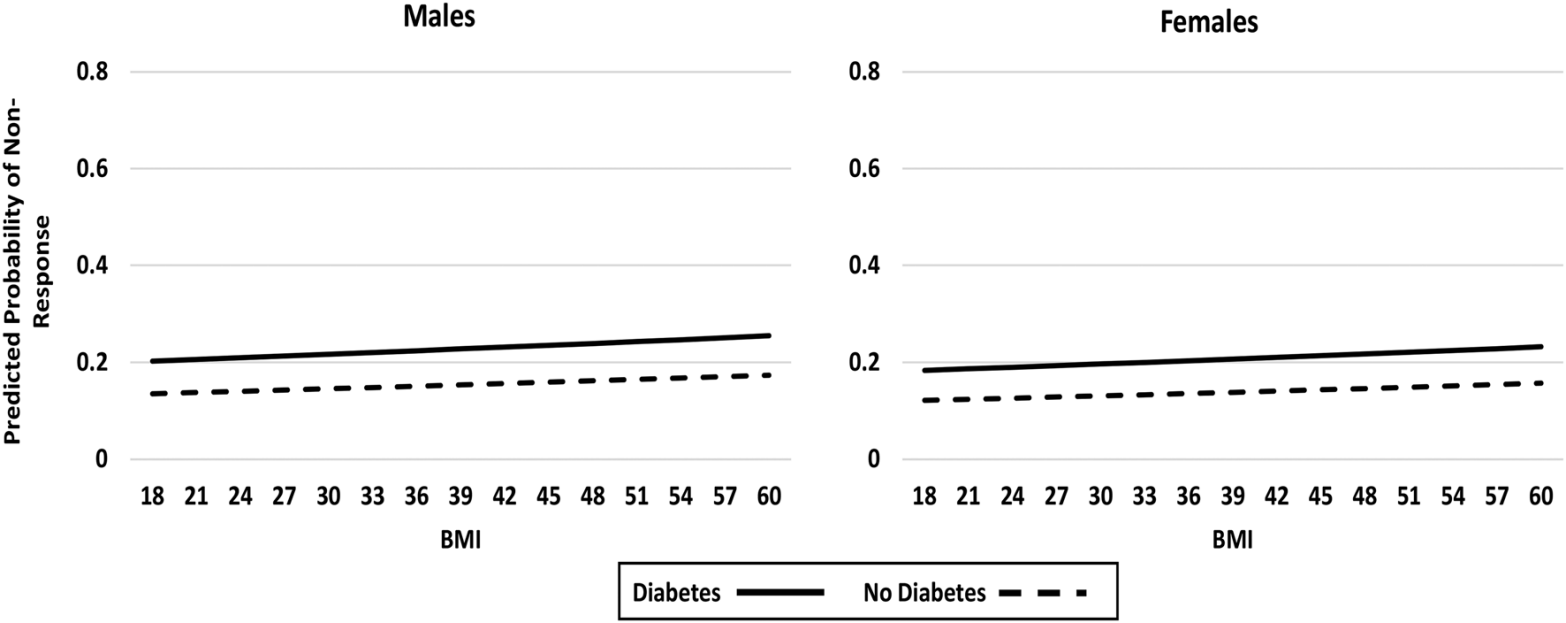
Impact of diabetes status, sex and BMI on post-surgical pain non-response (< 30% improvement in WOMAC pain); Model 3—no interaction (showing no significant effects by sex, BMI or diabetes status).

**Figure 4:**
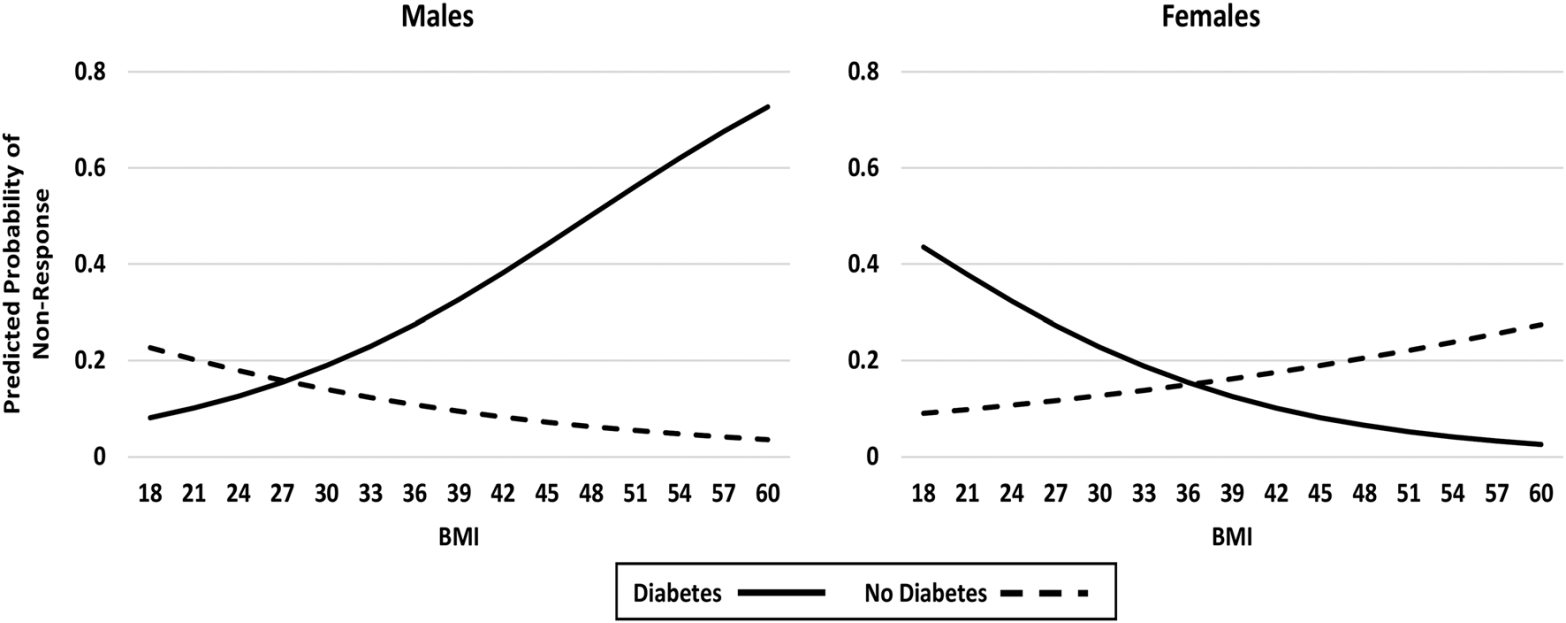
Impact of diabetes status, sex and BMI on post-surgical pain non-response (< 30% improvement in WOMAC pain): Model 4—with interactions (showing differential diabetes effects by sex and BMI).

Similar results were found when WOMAC pain non-response was investigated (Table 4). Diabetes was not initially a significant independent predictor of non-response (Model 3 and Fig. 3), but with the addition of the three way interaction (Model 4), diabetes was significantly associated with non-response (p = 0.006). As depicted in Fig. 4, there was a sharp increase in the probability of surgical non-response with increasing BMI for males, but a decrease in probability of non-response for females.

**Table 4:** Predictors of non-response (< 30% improvement) in WOMAC pain at 12 months post-TJR.

Variable	Model 3: without interaction*		Model 4: with interaction** (DIABETES*BMI*SEX)	
	Odds Ratio (95% CI)	p-value	Odds Ratio (95% CI)	p-value
Surgical site (Hip vs. Knee)	**0.27 (0.16, 0.44)**	** < 0.001**	**0.27 (0.17, 0.45)**	** < 0.001**
Sex (Female vs. Male)	0.88 (0.59, 1.34)	0.558	**	**0.045**
Age	0.98 (0.96, 1.01)	0.138	0.98 (0.96, 1.01)	0.141
Education (Post-secondary vs. ≤ High school)	0.75 (0.49, 1.15)	0.189	0.72 (0.47, 1.11)	0.134
BMI	1.01 (0.97, 1.04)	0.701	**	0.182
Comorbidity count	1.03 (0.90, 1.18)	0.699	1.03 (0.90, 1.18)	0.684
Symptomatic joint count	1.03 (0.97, 1.10)	0.337	1.03 (0.97, 1.10)	0.319
Depressive symptoms score (0 to 21)	0.97 (0.90, 1.05)	0.454	0.97 (0.90, 1.05)	0.447
Anxiety symptoms score (0 to 21)	10.5 (0.98, 1.12)	0.145	1.05 (0.98, 1.12)	0.150
WOMAC pain score (0 to 20)	**0.82 (0.73, 0.91)**	** < 0.001**	**0.82 (0.74, 0.92)**	** < 0.001**
WOMAC physical function score (0 to 68)	1.03 (1.00, 1.06)	0.088	1.03 (0.99, 1.06)	0.137
Opioid Use (Daily or occasional vs. No use)	1.22 (0.77, 1.93)	0.402	1.23 (0.77, 1.96)	0.381
Neuropathic pain score (-1 to 38) (Likely vs. Unlikely/possibly)	**2.54 (1.40, 4.61)**	**0.002**	**2.56 (1.41, 4.67)**	**0.002**
Diabetes (present vs. absent)	1.63 (0.95, 2.78)	0.074	**	0.081
Diabetes*BMI	–	–	**	**0.036**
Diabetes*Sex	–	–	**	**0.008**
Sex*BMI	–	–	**	**0.053**
Diabetes*Sex*BMI	–	–	**	**0.006**

*See Fig. 3 (Model 3, without interaction) for depiction of the effect of diabetes, sex and BMI.

**To interpret interactions, please refer to Fig. 4 (Model 4, with interaction) for depiction of the effects of diabetes, sex and BMI.

Findings were also consistent when the alternative definitions of surgical non-response were considered in the supplemental/sensitivity analyses. The effect of diabetes similarly varied by sex and BMI with non-response based on joint-specific WOMAC MCID values (for physical function: p = 0.046, Table S3 and Figs. S5 and S6; for pain: p = 0.022, Table S4 and Figs. S6 and S7), and non-response based on OARSI-OMERACT responder criteria (p = 0.029, Table S5, and Figs. S9 and S10). In all cases, the direction of the association between diabetes and outcome differed for males and females.

In analyses where waist circumference was used instead of BMI, findings were unchanged; the effect of diabetes varied by sex and waist circumference (for physical function: p = 0.032, Table S1 and Figs. S1 and S2; for pain: p = 0.001, Table S2 and Figs. S3 and S4).

Finally, there were some similarities in terms of the significance of the control covariates across the main and supplemental models. Across all models, hip patients had significantly lower odds of surgical non-response than knee patients, and those with likely neuropathic pain symptoms had higher odds than those without. Also, worse baseline pain and physical function scores were associated with a decreased likelihood of non-response on the corresponding outcome. With some variation in magnitude of associations across models, worse mental health scores were also significantly associated with increased odds of surgical non-response. Depressive symptoms scores was a significant covariate in the main physical function outcome model (Table 3), while anxiety was significant in the three supplemental models in which alternative definitions of outcome were considered. (Tables S3, S4, S5) A higher number of additional symptomatic joints was also significantly associated with increased odds of surgical non-response in multiple models.

## Discussion

Diabetes is an increasingly common comorbidity in TJA patients. However, its impact on surgical patient-reported outcomes has been unclear due to conflicting findings in the literature. Our study of a large cohort of TJA patients initially found no impact of diabetes on either 12-month post-surgical pain or physical function response. However, when we considered that the effect of diabetes may vary by sex and BMI, a significant diabetes effect was identified. In males with diabetes, the probability of a poor TJA outcome increased with increasing BMI, whereas in females with diabetes, the probability of a poor TJA outcome decreased with increasing BMI. These findings suggest that a simple consideration of diabetes as present vs. absent is insufficient to understand the impact of diabetes, and requires an explicit consideration of sex differences in its effects, by BMI. This has potential implications for future research, and for possible interventions and patient education for this large and growing clinical OA population undergoing TJA.

The findings should not be interpreted as suggesting that high BMI is unimportant in female TJA patients with diabetes. Obesity affects almost every organ system in the body and is associated with numerous negative health impacts, regardless of sex^35^. In addition, obesity can be associated with more technically challenging TJA and higher rates of complications such as infections, longer hospital stays and higher rates of revision^36^. Our findings suggest that diabetes may increase the risk of a poor TJA pain or physical function outcome for males and females, but that this is of greatest concern at different levels of BMI for males (higher BMI) and females (lower BMI).

To date, studies investigating the impact of diabetes on patient-reported outcomes after TJA have produced varying findings^10–14^. The specific outcomes considered have varied, with some considering only generic, rather than OA-specific, outcomes, and in many cases post-surgical status scores are examined without consideration of pre-surgical scores. Furthermore, there have been variable degrees of control for other patient factors. Brock et al.^13^ examined the impact of diabetes on WOMAC change scores at one-year after surgery using multivariable regression models. They did not identify a significant association between diabetes and pain scores, but did find that patients with diabetes had smaller improvements in physical function, particularly for those with inadequate pre-surgical glycemic control. King et al.^14^ examined the impact of diabetes on a number of 12-month knee TJA outcomes, including WOMAC pain and KOOS (Knee injury and Osteoarthritis Outcome Score Short Form) physical function. They examined both 12-month status and change scores and did not find significant associations with comorbid diabetes in multivariable regression analyses. However, diabetes was associated with 36% lower odds of reporting a 12-month Patient Acceptable Symptom State (PASS). Lenguerrad et al.^12^ found that there was no impact of diabetes on WOMAC pain and function status scores 12-months after surgery once they adjusted for BMI and number of comorbidities. Zhang et al.^10^ similarly found no association with 12-month WOMAC total scores. To the best of our knowledge, our study is the first to consider sex and BMI as effect modifying variables, rather than as adjustment or control variables, in analyses examining diabetes impact on patient-reported outcomes.

Sex differences in diabetes and obesity are widely recognized in the literature and the importance of their consideration in clinical care and research has been highlighted^18,19,37^. Our contrasting findings for men and women with OA are not unprecedented. Using data from the MOST study, Rogers-Soeder et al.^38^ found that insulin resistance was inversely associated with incident radiographic knee OA in women, but not in men. In the previously cited NHANES-based work, sex- and body-size- specific associations between insulin resistance and knee OA were reported, such that in obese women insulin resistance was inversely related to OA. Although these studies focussed on OA occurrence, and not TJA outcomes, they similarly identified inverse relationships with diabetes measures in women that appear somewhat counter-intuitive.

OA, obesity and diabetes are known to be associated with pro-inflammatory states^39,40^. There have been a number of interesting findings reported in the literature in which associations of various inflammatory markers or cytokines with symptoms in OA have differed by sex. For example, Perruccio et al. reported different associations between systemic adipokines (largely fat-derived chemokines) and OA joint burden for women and for men with hip and knee OA^41^. In additional work, they demonstrated that increasing levels of systemic C-reactive protein (CRP; a marker of inflammation) were associated with greater joint burden in women, but not in men^42^ and that the association of specific inflammatory markers with OA pain varied by sex^43^, including relationships for some markers that were in opposite directions for men and women. It is unclear if similar relationships with specific inflammatory markers may underlie the sex- and BMI- specific associations we identified for TJA outcomes in OA patients with diabetes. Future work should consider inflammatory and other metabolic parameters to further clarify sex-specific relationships with diabetes in OA.

We identified notable associations between some of the other considered variables in the study and an increased risk of a non-response following TJA. Across all models, pre-surgical neuropathic-like pain scores were associated with an increased risk of non-response, regardless of how outcome was defined. In our primary physical function and pain analyses, having ‘likely’ neuropathic pain was associated with an approximately 2.5 times greater odds of reporting a less than 30% improvement in pain or physical function by 12 months after surgery. There has been increasing recognition of the potential for pain sensitization in OA^44^ and neuropathic-like pain symptoms have been reported to be associated with a poor TJA outcome in the limited available research^45^. Despite this, medications that specifically target neuropathic pain are not commonly considered in this population^45,46^. We also identified significant associations between measures of poorer mental health, either depressive or anxiety symptoms, and increased risk of a poor surgical outcome. We have previously reported on the discrepancy between rates of ‘caseness’ of depression and depression diagnosis and treatment in our TJA cohort^47^. Taken together, findings highlight the need for appropriate mental health screening and treatment for TJA patients.

Strengths of our study include its relatively large sample size and multivariable adjustment for a range of patient factors. Our primary outcome variables were based on percentage change scores; the importance of accounting for pre-surgical scores is increasingly being recognized in the literature^8^. Nonetheless, our findings were consistent across sensitivity analyses that considered alternative outcome definitions, incorporating varying degrees of percentage and absolute change based on OARSI-OMERACT criteria^34^, as well as published joint-specific MCID criteria for absolute change^28,29^. Findings were also consistent when we considered waist circumference instead of body mass index as a measure of body size. There is a large body of literature that discusses the relative strengths and weaknesses of a number of measures of body size^48^. The main criticisms of body mass index include that it does not adequately measure body fat or account for body fat distribution. It has a high specificity but lower sensitivity for detecting obesity and may be less reliable in elderly populations^49^. It is not clear what the most appropriate measure of body composition would be in the context of studying diabetes in an OA population. Regardless, in future work it may be informative to explore alternative measures of body size and the implications for identifying sex-specific effects. We identified patients with diabetes based on self-report. Although the accuracy of self-reported diabetes has been reported to be high^50^, some patients may have been misclassified. As it is less likely that a patient would indicate they have diabetes when this is not the case, rather than having diabetes and not reporting it, misclassification may have resulted in reduced magnitudes of associations between diabetes and outcomes. We were also unable to identify diabetes type. Given the relative prevalence of Type 1 and Type 2 diabetes in the population, it is likely that our findings are most generalizable to patients with Type 2 diabetes. We also did not have data on duration of diabetes or access to measures of diabetes control (e.g. blood glucose, HbA1c) for the cohort, which also may be relevant to consider in future studies. Although we did control for an extensive list of potentially important control variables in our regression models, the possibility of residual confounding effects must be considered. It may also be relevant to examine differences in the impact of diabetes on TJA outcomes by race. We were limited in our ability to consider this, as approximately 85% of our sample indicated they were ‘White’. We also did not have data on the use of hormonal replacement medications and could not examine their potential impact.

These novel findings are suggestive of important differences in diabetes impact by sex and body size in this clinical OA population. This may explain some of the variability in findings across studies that have examined the impact of diabetes on TJA outcomes but not considered the simultaneous effects of sex and body composition. The findings warrant confirmation in additional work with larger sample sizes to more comprehensively examine sex- and body composition-specific effects. This may not only improve our understanding of the relationship between OA, diabetes and body composition which may be different for males and females, but the work also has the potential to inform patient management, patient education and decision-making for these high volume surgical interventions in OA.

### Supplementary Information

Supplementary Information.

## Data Availability

The data underlying the findings of this study contain potentially identifying and sensitive patient information and cannot be shared publicly. Readers can request access to the data by completing a data request following an Arthritis Program Research Committee and UHN Ethics Committee approved research proposal and a data access agreement, signed by all parties (contact via Christian Veillette; Christian.Veillette@uhn.ca).
