# Supplementary material for: The impact of diabetes status on pain and physical function following total joint arthroplasty for hip and knee osteoarthritis: variation by sex and body mass index
Source: Sci Rep. 2024 May 15;14:11152. doi: 10.1038/s41598-024-61847-0 (PMC11096302; doi:10.1038/s41598-024-61847-0)
Supplement: Supplementary file 1 — Supplementary Information. [file 41598_2024_61847_MOESM1_ESM.docx]

**SUPPLEMENTAL DATA:** *The Impact of Diabetes Status on Pain and Physical Function Following Total Joint Arthroplasty for Hip and Knee Osteoarthritis: Variation By Sex and Body Mass Index*

**Authors:** J. Denise Power, Anthony V. Perruccio, Mayilee Canizares, J. Roderick Davey, Rajiv Gandhi, Nizar N. Mahomed, Khalid Syed, Christian Veillette, Y.Raja Rampersaud

**Table S1 – Predictors of non-response (<30% change in WOMAC physical function) at 12 months post-TJR, using waist circumference instead of BMI as a covariate**

|  | **Model S1: without interaction^*^** | | **Model S2: with interaction^**^**  **(DIABETES*WAIST CIRCUMFERENCE*SEX)** | |
| --- | --- | --- | --- | --- |
| **Variable** | **Odds Ratio (95% CI)** | **p-value** | **Odds Ratio (95% CI)** | **p-value** |
| **Surgical site**  (Hip vs. Knee) | **0.38**  **(0.25, 0.59)** | **<0.001** | **0.39**  **(0.25, 0.61)** | **<0.001** |
| **Sex**  (Female vs. Male) | 0.81  (0.54, 1.22) | 0.313 | ** | 0.789 |
| **Age** | 1.01  (0.99, 1.03) | 0.405 | 1.01  (0.99, 1.03) | 0.344 |
| **Education**  (Post-secondary vs.  ≤High school) | 0.74  (0.50, 1.11) | 0.145 | 0.74  (0.49, 1.11) | 0.143 |
| **Waist circumference** | 1.01  (0.99, 1.03) | 0.079 | ****** | 0.260 |
| **Comorbidity Count** | 0.99  (0.88, 1.12) | 0.898 | 0.98  (0.87, 1.11) | 0.756 |
| **Symptomatic joint count** | 1.06  (1.00, 1.12) | 0.068 | 1.06  (1.00, 1.12) | 0.074 |
| **Depressive Symptoms score**  **(0 to 21)** | 1.06  (0.99, 1.14) | 0.073 | **1.07**  **(1.00, 1.15)** | **0.052** |
| **Anxiety Symptoms score**  **(0 to 21)** | 1.03  (0.97, 1.09) | 0.375 | 1.03  (0.94, 1.15) | 0.404 |
| **WOMAC pain score**  **(0 to 20)** | 1.03  (0.93, 1.14) | 0.530 | 1.04  (0.94, 0.99) | 0.479 |
| **WOMAC physical function score**  **(0 to 68)** | **0.96**  **(0.93, 0.99)** | **0.012** | **0.96**  **(0.93, 0.99)** | **0.007** |
| **Opioid Use**  **(**Daily or occasional vs.  No use) | 0.87  (0.56, 1.35) | 0.538 | 0.88  (0.57, 1.38) | 0.593 |
| **Neuropathic pain score** **(-1 to 38)**  (Likely vs. Unlikely/possibly) | **2.46**  **(1.42, 4.26)** | **0.001** | **2.44**  **(1.41, 4.23)** | **0.001** |
| **Diabetes**  **(present vs. absent)** | 1.00*  (0.58, 1.72) | 0.991 | ** | 0.212 |
| **Diabetes* Waist circumference** | - | - | ** | 0.265 |
| **Diabetes*Sex** | - | - | ** | **0.022** |
| **Sex* Waist circumference** | - | - | ** | 0.942 |
| **Diabetes*Sex* Waist circumference** | - | - | ** | **0.032** |

*See Figure S1 (Model S1, without interaction) for depiction of the effect of diabetes, sex and waist circumference

**To interpret interactions, please refer to Figure S2 (Model S2, with interaction) for depiction of the effects of diabetes, sex and waist circumference.

**Figure S1: Impact of diabetes status, sex and waist circumference on post-surgical physical function non-response (<30% change in WOMAC physical function): Model S1 - no interaction (showing no significant effects by sex, waist circumference or diabetes status)**

**Figure S2: Impact of diabetes status, sex and waist circumference on post-surgical physical function non-response (<30% change in WOMAC physical function): Model S2 - with interaction (showing differential diabetes effects by sex and waist circumference)**

**Table S2: Predictors of non-response (<30% change in WOMAC pain) at 12 months post-TJR, using waist circumference instead of BMI as a covariate**

|  | **Model S3: without interaction^*^** | | **Model S4: with interaction^**^**  **(DIABETES*WAIST CIRCUMFERENCE*SEX)** | |
| --- | --- | --- | --- | --- |
| **Variable** | **Odds Ratio (95% CI)** | **p-value** | **Odds Ratio (95% CI)** | **p-value** |
| **Surgical site**  (Hip vs. Knee) | **0.28**  **(0.17, 0.46)** | **<0.001** | **0.29**  **(0.18, 0.48)** | **<0.001** |
| **Sex**  (Female vs. Male) | 0.94  (0.61, 1.44) | 0.760 | ** | 0.158 |
| **Age** | 0.99  (0.96, 1.01) | 0.222 | 0.99  (0.96, 1.01) | 0.237 |
| **Education**  (Post-secondary vs.  ≤High school) | 0.81  (0.52, 1.24) | 0.331 | 0.79  (0.51, 1.22) | 0.278 |
| **Waist circumference** | 1.01  (0.99, 1.02) | 0.311 | ****** | 0.736 |
| **Comorbidity Count** | 1.01  (0.88, 1.16) | 0.912 | 1.02  (0.89, 1.18) | 0.746 |
| **Symptomatic joint count** | 1.03  (0.97, 1.10) | 0.369 | 1.03  (0.97,1.10) | 0.380 |
| **Depressive Symptoms score**  **(0 to 21)** | 0.98  (0.91, 1.06) | 0.558 | 0.98  (0.91, 1.06) | 0.628 |
| **Anxiety Symptoms score**  **(0 to 21)** | 1.05  (0.99, 1.12) | 0.137 | 1.05  (0.98, 1.12) | 0.175 |
| **WOMAC pain score**  **(0 to 20)** | **0.82**  **(0.73, 0.92)** | **<0.001** | **0.82**  **(0.73, 0.92)** | **<0.001** |
| **WOMAC physical function score**  **(0 to 68)** | 1.03  (1.00, 1.06) | 0.090 | 1.02  (0.99, 1.06) | 0.167 |
| **Opioid Use**  **(**Daily or occasional vs.  No use) | 1.25  (0.79, 1.98) | 0.346 | 1.32  (0.83, 2.10) | 0.246 |
| **Neuropathic pain score** **(-1 to 38)**  (Likely vs. Unlikely/possibly) | **2.53**  **(1.39, 4.62)** | **0.003** | **2.60**  **(1.42, 4.78)** | **0.002** |
| **Diabetes**  **(present vs. absent)** | 1.63  (0.95, 2.79) | 0.078 | ** | 0.189 |
| **Diabetes* Waist circumference** | - | - | ** | 0.127 |
| **Diabetes*Sex** | - | - | ** | **0.001** |
| **Sex* Waist circumference** | - | - | ** | 0.159 |
| **Diabetes*Sex* Waist circumference** | - | - | ** | **0.001** |

*See Figure S3 (Model S3, without interaction) for depiction of the effect of diabetes, sex and waist circumference

**To interpret interactions, please refer to Figure S4 (Model S4, with interaction) for depiction of the effects of diabetes, sex and waist circumference.

**Figure S3: Impact of diabetes status, sex and waist circumference post-surgical pain non-response (<30% change in WOMAC pain): Model S3 - no interaction (showing no significant effects by sex, waist circumference or diabetes status)**

**Figure S4: Impact of diabetes status, sex and waist circumference on post-surgical pain non-response (<30% change in WOMAC pain): Model S4 - with interaction (showing differential diabetes effects by sex and waist circumference)**

**Table S3 – Predictors of non-response (not achieving MCID in joint-specific MCID for WOMAC physical function) at 12 months post-TJR**

|  | **Model S5: without interaction^*^** | | **Model S6: with interaction^**^**  **(DIABETES*BMI*SEX)** | |
| --- | --- | --- | --- | --- |
| **Variable** | **Odds Ratio (95% CI)** | **p-value** | **Odds Ratio (95% CI)** | **p-value** |
| **Surgical site**  (Hip vs. Knee) | **0.38**  **(0.27, 0.54)** | **<0.001** | **0.38**  **(0.27, 0.54)** | **<0.001** |
| **Sex**  (Female vs. Male) | 0.77  (0.56, 1.07) | 0.120 | ** | 0.478 |
| **Age** | 1.01  (0.99, 1.03) | 0.166 | 1.02  (1.00, 1.04) | 0.143 |
| **Education**  (Post-secondary vs.  ≤High school) | 0.89  (0.62, 1.26) | 0.494 | 0.87  (0.61, 1.23) | 0.430 |
| **BMI** | 1.03  (1.00, 1.06) | 0.078 | ** | 0.398 |
| **Comorbidity Count** | 1.02  (0.92, 1.13) | 0.660 | 1.02  (0.92, 1.14) | 0.664 |
| **Symptomatic joint count** | **1.08**  **(1.02,1.14)** | **0.006** | **1.08**  **(1.03, 1.14)** | **0.006** |
| **Depressive Symptoms score**  **(0 to 21)** | 1.05  (0.98, 1.11) | 0.146 | 1.05  (0.99, 1.11) | 0.126 |
| **Anxiety Symptoms score**  **(0 to 21)** | **1.08**  **(1.03, 1.14)** | **0.003** | **1.08**  **(1.03, 1.14)** | **0.004** |
| **WOMAC pain score**  **(0 to 20)** | 0.97  (0.89, 1.06) | 0.531 | 0.98  (0.90, 1.07) | 0.643 |
| **WOMAC physical function score**  **(0 to 68)** | **0.92**  **(0.90, 0.94)** | **<0.001** | **0.92**  **(0.89, 0.94)** | **<0.001** |
| **Opioid Use**  **(**Daily or occasional vs.  No use) | 0.87  (0.59, 1.28) | 0.478 | 0.88  (0.60, 1.29) | 0.505 |
| **Neuropathic pain score** **(-1 to 38)**  (Likely vs. Unlikely/possibly) | **2.22**  **(1.31, 3.74)** | **0.003** | **2.20**  **(1.30, 3.72)** | **0.003** |
| **Diabetes**  **(present vs. absent)** | 1.09  (0.67, 1.75) | 0.733 | ** | 0.647 |
| **Diabetes*BMI** | - | - | ** | 0.504 |
| **Diabetes*Sex** | - | - | ** | 0.070 |
| **Sex*BMI** | - | - | ** | 0.618 |
| **Diabetes*Sex*BMI** | - | - | ** | **0.046** |

*See Figure S5 (Model S5, without interaction) for depiction of the effect of diabetes, sex and BMI

**To interpret interactions, please refer to Figure S6 (Model S6, with interaction) for depiction of the effects of diabetes, sex and BMI.

**Figure S5: Impact of diabetes status, sex and BMI on post-surgical physical function non-response (not achieving joint-specific MCID for WOMAC physical function): Model S5 -no interaction (showing no significant effects by sex, BMI or diabetes status)**

**Figure S6: Impact of diabetes status, sex and BMI on post-surgical physical function non-response (not achieving joint-specific MCID for WOMAC physical function): Model S6 - with interaction (showing differential diabetes effects by sex and BMI)**

**Table S4 – Predictors of non-response (not achieving joint-specific MCID for WOMAC pain) at 12 months post-TJR**

|  | **Model S7: without interaction^*^** | | **Model S8: with interaction^**^**  **(DIABETES*BMI*SEX)** | |
| --- | --- | --- | --- | --- |
| **Variable** | **Odds Ratio (95% CI)** | **p-value** | **Odds Ratio (95% CI)** | **p-value** |
| **Surgical site**  (Hip vs. Knee) | **0.29**  **(0.20, 0.41)** | **<0.001** | **0.29**  **(0.20, 0.42)** | **<0.001** |
| **Sex**  (Female vs. Male) | 0.87  (0.63, 1.22) | 0.426 | ** | 0.062 |
| **Age** | 1.00  (0.98, 1.02) | 0.891 | 1.00  (0.98, 1.02) | 0.873 |
| **Education**  (Post-secondary vs.  ≤High school) | 0.98  (0.69, 1.41) | 0.921 | 0.96  (0.67, 1.38) | 0.820 |
| **BMI** | 1.01  (0.98, 1.05) | 0.356 | ** | 0.324 |
| **Comorbidity Count** | 0.98  (0.89, 1.09) | 0.717 | 0.98  (0.88, 1.09) | 0.688 |
| **Symptomatic joint count** | **1.06**  **(1.01, 1.12)** | **0.034** | **1.06**  **(1.01, 1.12)** | **0.030** |
| **Depressive Symptoms score**  **(0 to 21)** | 1.05  (0.99, 1.12) | 0.107 | 1.05  (0.99, 1.12) | 0.122 |
| **Anxiety Symptoms score**  **(0 to 21)** | **1.07**  **(1.01, 1.13)** | **0.016** | **1.07**  **(1.01, 1.13)** | **0.015** |
| **WOMAC pain score**  **(0 to 20)** | **0.70**  **(0.64, 0.77)** | **<0.001** | **0.70**  **(0.64, 0.77)** | **<0.001** |
| **WOMAC physical function score**  **(0 to 68)** | 1.02  (1.00, 1.05) | 0.101 | 1.02  (0.99, 1.05) | 0.137 |
| **Opioid Use**  **(**Daily or occasional vs.  No use) | 0.97  (0.65, 1.44) | 0.873 | 0.97  (0.65, 1.45) | 0.889 |
| **Neuropathic pain score** **(-1 to 38)**  (Likely vs. Unlikely/possibly) | **2.24**  **(1.30, 3.85)** | **0.004** | **2.23**  **(1.29, 3.84)** | **0.004** |
| **Diabetes**  **(present vs. absent)** | 1.39  (0.86, 2.24) | 0.178 | ** | 0.074 |
| **Diabetes*BMI** | - | - | ** | **0.036** |
| **Diabetes*Sex** | - | - | ** | **0.031** |
| **Sex*BMI** | - | - | ** | 0.076 |
| **Diabetes*Sex*BMI** | - | - | ** | **0.022** |

*See Figure S7 (Model S7, without interaction) for depiction of the effect of diabetes, sex and BMI

**To interpret interactions, please refer to Figure S8 (Model S8, with interaction) for depiction of the effects of diabetes, sex and BMI.

**Figure S7: Impact of diabetes status, sex and BMI on post-surgical pain non-response (not achieving joint-specific MCID for WOMAC pain): Model S7 -no interaction (showing no significant effects by sex, BMI or diabetes status)**

**Figure S8: Impact of diabetes status, sex and BMI on post-surgical physical function non-response (not achieving joint-specific MCID for WOMAC pain): Model S8 - with interaction (showing differential diabetes effects by sex and BMI)**

**Table S5 – Predictors of non-response (not meeting OARSI-OMERACT responder criteria) at 12 months post-TJR**

|  | **Model S9: without interaction^*^** | | **Model S10: with interaction^**^**  **(DIABETES*BMI*SEX)** | |
| --- | --- | --- | --- | --- |
| **Variable** | **Odds Ratio (95% CI)** | **p-value** | **Odds Ratio (95% CI)** | **p-value** |
| **Surgical site**  (Hip vs. Knee) | **0.35**  **(0.20, 0.60)** | **<0.001** | 0.35  (0.20, 0.61) | **<0.001** |
| **Sex**  (Female vs. Male) | 0.84  (0.53, 1.34) | 0.470 | ** | **0.029** |
| **Age** | 1.00  (0.97, 1.02) | 0.772 | 1.00  (0.97, 1.03) | 0.908 |
| **Education**  (Post-secondary vs.  ≤High school) | 0.80  (0.50, 1.30) | 0.365 | 0.76  (0.47, 1.24) | 0.268 |
| **BMI** | 1.02  (0.98, 1.06) | 0.277 | ** | 0.311 |
| **Comorbidity Count** | 1.07  (0.94, 1.22) | 0.319 | 1.04  (0.91, 1.19) | 0.534 |
| **Symptomatic joint count** | 1.03  (0.96, 1.11) | 0.393 | 1.04  (0.96, 1.11) | 0.356 |
| **Depressive Symptoms score**  **(0 to 21)** | 1.01  (0.93, 1.10) | 0.825 | 1.01  (0.93, 1.10) | 0.803 |
| **Anxiety Symptoms score**  **(0 to 21)** | **1.08**  **(1.01, 1.16)** | **0.035** | **1.09**  **(1.01, 1.17)** | **0.024** |
| **WOMAC pain score**  **(0 to 20)** | 0.90  (0.79, 1.01) | 0.071 | 0.90  (0.80, 1.02) | 0.099 |
| **WOMAC physical function score**  **(0 to 68)** | **0.96**  **(0.93, 0.99)** | 0.016 | **0.96**  **(0.92, 0.99)** | **0.010** |
| **Opioid Use**  **(**Daily or occasional vs.  No use) | 1.39  (0.83, 2.33) | 0.214 | 1.37  (0.81, 2.32) | 0.239 |
| **Neuropathic pain score** **(-1 to 38)**  (Likely vs. Unlikely/possibly) | **3.84**  **(1.97, 7.49)** | **<0.001** | **3.81**  **(1.95, 7.46)** | **<0.001** |
| **Diabetes**  **(present vs. absent)** | 0.94  (0.49, 1.82) | 0.859 | ** | 0.013 |
| **Diabetes*BMI** | - | - | ** | **0.012** |
| **Diabetes*Sex** | - | - | ** | **0.002** |
| **Sex*BMI** | - | - | ** | **0.045** |
| **Diabetes*Sex*BMI** | - | - | ** | **0.029** |

*See Figure S9 (Model S9, without interaction) for depiction of the effect of diabetes, sex and BMI

**To interpret interactions, please refer to Figure S10 (Model S10, with interaction) for depiction of the effects of diabetes, sex and BMI.

**Figure S9: Impact of diabetes status, sex and BMI on post-surgical non-response (not meeting OARSI-OMERACT responder criteria): Model S9 -no interaction (showing no significant effects by sex, BMI or diabetes status)**

**Figure S10: Impact of diabetes status, sex and BMI on post-surgical non-response (not meeting OARSI-OMERACT responder criteria): Model S10 - with interaction (showing differential diabetes effects by sex and BMI)**
